# Efficacy of hydroxychloroquine in hand osteoarthritis: A protocol for systematic review and meta-analysis of randomized clinical trial

**DOI:** 10.1097/MD.0000000000023517

**Published:** 2020-12-11

**Authors:** Qiang-Qiang Li, Ya-Dong Xie, Wen-Qiang Liang, Guo-Qing Yang, Huai-Bin Zhang, Yong-Ping Wang

**Affiliations:** a The First Clinical Medical College of Lanzhou University; b Department of Orthopedics, The First Hospital of Lanzhou University, Lanzhou City, Gansu Province, China.

**Keywords:** hand function, hydroxychloroquine, osteoarthritis, pain

## Abstract

**Background:**

Symptoms of hand osteoarthritis result in activity limitations and lower quality of life. Hydroxychloroquine, which has been used successfully in the treatment of many autoimmune diseases, can suppress inflammation and might also be beneficial in hand osteoarthritis.

**Methods:**

We plan to perform a systematic review and meta-analysis of randomized clinical trial to determine the symptom-modifying effect of hydroxychloroquine in hand osteoarthritis. We will search PubMed, EMBASE, Cochrane Library, and Web of Science using a comprehensive strategy. The related conference proceedings and reference lists of the included studies will also be checked to identify additional studies. Two reviewers will screen retrieved records, extract information and assess the risk of bias independently. Stata v15.1 software will be used to conduct data synthesis.

**Results:**

This study will be submitted to a peer-reviewed journal for publication.

**Conclusion:**

We hope it will provide a relatively comprehensive reference for clinical practice and future relevant clinical trials.

**INPLASY registration number:**

INPLASY2020110005

## 1 Introduction

As a common form of osteoarthritis (OA),^[1]^ symptomatic hand osteoarthritis with an increased prevalence of 26% in women and 13% in men ages over 70 years,^[2]^ include pain and stiffness and lead to reduced hand mobility and grip strength, resulting in activity limitations and lower quality of life.^[3]^ Besides hand OA has a high burden of disease and an unmet medical need for effective therapeutic options.^[4]^

Current symptomatic treatment of hand OA consists of non-pharmacological, including education and exercise, and pharmacologic approaches, including paracetamol, topical non-steroidal anti-inflammatory drugs, topical capsaicin, chondroitin sulfate, and intraarticular corticosteroids.^[5–7]^ But these therapies are modest because their effects are small and inconsistent, and long-term effectiveness is not investigated.

Hydroxychloroquine (HCQ) has been used clinically in the treatment of mild rheumatoid arthritis and other autoimmune diseases by the suppression of inflammation for many years. Hand OA involves not only mechanical triggers but also local inflammation caused pain, stiffness, reduced motion, and radiographical damage progression.^[8,9]^ Therefore, inflammation is a potential treatment target and HCQ might also be beneficial in hand OA.^[10]^

Heretofore, there have only been a few trials that have proven some beneficial effect of HCQ in hand OA^[11,12]^ and HCQ is not recommended in guidelines.^[13]^ To further confirm the efficacy of HCQ in hand OA, we performed this systematic review and meta-analysis to explore the clinical outcomes.

## 2 Methods

### 2.1 Protocol registration

This protocol was registered with the International Platform of Registered Systematic Review and Meta-Analysis Protocols (INPLASY). The registration number is INPLASY2020110005 (https://inplasy.com//). The content of this protocol will follow the preferred reporting items for systematic review and meta-analysis protocols recommendations.^[14]^ We also plan to conduct it in accordance with the Cochrane Handbook for the Systematic Reviews of Interventions and preferred reporting items for systematic review and meta-analysis guidelines.^[15]^

### 2.2 Eligibility criteria

#### 2.2.1 Types of studies

Randomized controlled trial (RCT) without published year, publication status limitations.

#### 2.2.2 Types of participants

Inclusion criteria required patients ages with primary hand OA according to the American College of Rheumatology classification criteria.^[16]^ Exclusion criteria include secondary hand OA caused by inflammatory rheumatic diseases such as rheumatoid arthritis, psoriatic arthritis, peripheral arthritis, or spondyloarthropathy, use of HCQ within 3 months before enrolling the study, use of non-steroidal anti-inflammatory drugs or corticosteroids 7 days before enrolling the study, retinopathy, myasthenia gravis, or a known allergy or intolerance for HCQ.

#### 2.2.3 Types of interventions and comparators

1) The study group, treated with HCQ which range from 200 to 400 mg according to ideal body weight and
2) the control group, treated with placebo which had identical appearance to HCQ.

#### 2.2.4 Types of outcome measures

Outcomes were mainly identified by relevant literature and existed clinical practice. The primary outcome was self-reported hand pain in the previous 24 hours, measured on a100-mm visual analog scale at 6, 12, and 24 weeks from the beginning of the treatments. Secondary outcomes were self-reported hand pain and change in total score, compared to baseline, of the Australian Canadian hand osteoarthritis index^[17]^ and the Arthritis Impact Measurement Scale 2 short form at 6, 12, and 24 weeks from the beginning of the treatments.^[18]^ Besides, all the endpoints reported in the included studies will be collected and evaluated, although we may not mention some of them in this protocol.

### 2.3 Literature search

A systematic search of the literature will be conducted without language and year restrictions to identify all relevant RCT. We will search following electronic databases: PubMed, EMBASE, Cochrane Library and Web of Science from 2002 to Sep. 2020 using related search terms, including “hydroxychloroquine”, “hand osteoarthritis”, “Osteoarth”. In addition, congress and conference proceedings will be manually retrieved. Related articles and references of included research will also be tracked to find potential studies. If significant data was incomplete in included study, we will contact the authors to get unpublished data.

### 2.4 Study selection and data extraction

After imported into the Endnote X7 and duplication, retrieved records will be independently screened by 2 reviewers (QQL and YDX). Firstly, we will read the titles and abstracts of all identified records to exclude clearly unrelated records based on the inclusion criteria. Then the full texts of the articles retained were reviewed to further determine their suitability. Any disagreement will be resolved by a third reviewer (YPW and HBZ). We will show the selection process in details in the preferred reporting items for systematic review and meta-analysis flow chart.

Two authors (WQL and GQY) of this review will independently extract the data using a pre-defined form. The basic characteristics, related outcome and quality evaluation information of included studies will be collected. Similarly, any discrepancies will be resolved by a third reviewer (YPW and HBZ). Data extracted will include author, year, study type,number of participants, intervention, control, demographics, complications, previous history, pain visual analog scale, Australian Canadian hand osteoarthritis index and Arthritis Impact Measurement Scale 2 short format baseline and during the follow-up time.

### 2.5 Quality of evidence assessment

The quality of included studies will be assessed by grading of recommendations assessment development and evaluation, and divided into 4 levels: high quality, moderate quality, low quality, and very low quality.^[19]^

### 2.6 Assessment of study bias

Included study bias will be independently assessed by 2 reviewers (QQL and WQL) and any disagreement will be solved by a third reviewer (YPW and HBZ). For randomized controlled trials, we will use the Cochrane risk of bias tools to evaluate potential bias in 7 specific domains:

(1) sequence generation,
(2) allocation concealment,
(3) blinding of participants and personnel,
(4) blinding of outcome assessment,
(5) incomplete outcome data,
(6) selective outcome reporting,
(7) other bias.^[20]^

### 2.7 Statistical analysis

For dichotomous variables, the relative risk with 95% confidence intervals were calculated from each study. Continuous variables will be presented as standard mean difference with 95% confidence interval. All endpoints will be combined and performed meta-analysis by using DerSimonian and Laird random effects model.^[21]^ We assessed statistical heterogeneity by using Chi-squared test and I^2^ statistic. We will consider significant heterogeneity when *P* < .10 for Chi-squared test or I^2^ > 50%. All primary analyses were performed with STATA v15.1 (Stata Corp, College Station, TX).^[22]^

#### 2.7.1 Subgroup analysis

We will also conduct subgroup analysis to find more potential information based on pre-set criteria in different follow-up time.

#### 2.7.2 Sensitivity analysis

If the heterogeneity is high, we will conduct sensitivity analyses based on the patient age and follow-up time.

#### 2.7.3 Publication bias

The likelihood of publication bias was assessed graphically through the generation of funnel plots, evaluated using an Egger test.^[23]^

## 3 Results

The study does not require ethical approval because the meta-analysis are based on published research and the original data are anonymous. And this study will eventually be published in a peer-reviewed journal in the form of a scientific paper.

## 4 Discussion

Current symptomatic treatment of hand OA is limited, lack of efficacy of HCQ, and only been a few trials that have proven some beneficial effect of HCQ in hand OA. The results from our research may provide meaningful evidence for clinical practice and give a valuable reference for future study.

There seem to be some potential limitations for our study. Firstly, we only include English language articles, which might miss some important data in other language article. In addition, only RCT and no cohort studies will be included in our study, which may have an obstacle to our data pooling and results interpretation. But it probably help to promotes several more reliable conclusions and focus on more precious direction for future clinical studies to some extent. Notwithstanding its limitation, we hope to provide a prompt and credible evaluation for efficacy of HCQ in the treatment of hand OA.

## Author contributions

QQL and YDX conceived the idea for this study; QQL and GQY designed the meta-analysis; YDX and WQL provided statistical advice and input; QQL and YDX drafted the protocol; YPW and HBZ reviewed the protocol and provided critical feedback.

**Conceptualization:** Qiang-Qiang Li, Ya-Dong Xie.

**Data curation:** Ya-Dong Xie.

**Formal analysis:** Guo-Qing Yang.

**Methodology:** Qiang-Qiang Li, Ya-Dong Xie.

**Resources:** Wen-Qiang Liang.

**Writing – original draft:** Qiang-Qiang Li, Ya-Dong Xie.

**Writing – review & editing:** Huai-Bin Zhang, Yong-Ping Wang.
